# Machine learning based forecast for the prediction of inpatient bed demand

**DOI:** 10.1186/s12911-022-01787-9

**Published:** 2022-03-02

**Authors:** Manuel Tello, Eric S. Reich, Jason Puckey, Rebecca Maff, Andres Garcia-Arce, Biplab Sudhin Bhattacharya, Felipe Feijoo

**Affiliations:** 1 Pontificia Universidad Católica de Valparaíso, Valparaíso, Chile; 2 Geisinger Health System, Danville, PA USA

**Keywords:** Census, Overcrowding, Support vector machine, K-SVR

## Abstract

**Background:**

Overcrowding is a serious problem that impacts the ability to provide optimal level of care in a timely manner. High patient volume is known to increase the boarding time at the emergency department (ED), as well as at post-anesthesia care unit (PACU). Furthermore, the same high volume increases inpatient bed transfer times, which causes delays in elective surgeries, increases the probability of near misses, patient safety incidents, and adverse events.

**Objective:**

The purpose of this study is to develop a Machine Learning (ML) based strategy to predict weekly forecasts of the inpatient bed demand in order to assist the resource planning for the ED and PACU, resulting in a more efficient utilization.

**Methods:**

The data utilized included all adult inpatient encounters at Geisinger Medical Center (GMC) for the last 5 years. The variables considered were class of inpatient encounter, observation, or surgical overnight recovery (SORU) at the time of their discharge. The ML based strategy is built using the K-means clustering method and the Support Vector Machine Regression technique (K-SVR).

**Results:**

The performance obtained by the K-SVR strategy in the retrospective cohort amounts to a mean absolute percentage error (MAPE) that ranges between 0.49 and 4.10% based on the test period. Additionally, results present a reduced variability, which translates into more stable forecasting results.

**Conclusions:**

The results from this study demonstrate the capacity of ML techniques to forecast inpatient bed demand, particularly using K-SVR. It is expected that the implementation of this model in the workflow of bed capacity management will create efficiencies, which will translate in a more reliable, inexpensive and timely care for patients.

## Introduction

Designing and implementing effective hospital capacity management decisions and efficient staffing decisions is a critical challenge in every healthcare system. Specifically, a mismatch in bed capacity to bed demand and the corresponding clinical staffing requirements can have negative effects on key performance indicators like hospital access, wait times, quality of care as well as patient and employee satisfaction. It also invariably results in an increase of an assortment of costs. When the supply of hospital beds exceeds the demand of beds, it will likely result in higher costs and wasted resources from maintaining and staffing open beds [1, 2]. When the demand for beds exceeds the supply, the hospital will likely experience longer waiting times especially for patients in the ED who are waiting for an inpatient bed, which can result in sub-standard quality of care, poor employee satisfaction, increase rate of near-misses, and lower patient satisfaction [3–5].

The only independent variable in this phenomenon is the bed demand, which naturally fluctuates to express flu season, holidays, vacations, etc. Studying these changes in the demand for inpatient beds is a widely discussed and studied problem which impacts hospital’s ability to provide timely care for patients, among other negative effects such as increased probabilities of adverse events [6, 7], higher length of stay in the ED [8, 9], increased mortality [10], and low patient and staff satisfaction [11]. To tackle this issue, a myriad of strategies have been proposed and tested. For instance, the creation of holding units, the introduction of early discharge [12] and the adoption of surgical demand smoothing [13] were identified as effective approaches that can increase patient flow [14].

Many of these concepts include data insights provided by predictive modeling or ML approaches which relies on data from clinical admissions [15–17]. We recognize that providing near-term bed demand forecasts to administrative personnel such as operating room, schedulers, inpatient bed coordinators, and operations managers can increase their ability to assertively maintain efficient levels of occupancy.

While highly variable patient bed demand makes hospital capacity management even more challenging, the utilization of ML algorithms can help hospital operations stakeholders make better decisions by combining their insights with advanced analytics. There have been recent advances in ML techniques that are utilized in predictive analytics, outperforming traditional time series techniques (described in the literature review). This study leverages ML techniques and implements a strategy in the area of predictive analytics at a non-profit integrated healthcare system in Danville, Pennsylvania.

The objective of this study is to develop a ML based strategy able to provide an accurate forecast of the inpatient bed demand for the week. The proposed model will use tailored ML techniques to achieve a minimal error in the prediction, so the hospital’s capacity management team can take proactive measures, provided the best possible information, to ensure efficient patient flow, and therefore, outcomes.

## Background

This project takes place at Geisinger Medical Center (GMC), a hospital located in Danville, Pennsylvania and a part of Geisinger. GMC is a tertiary/quaternary teaching hospital with approximately 350 licensed and staffed adult inpatient beds. Since GMC is the only level 1 trauma center serving a large portion of the central Pennsylvania region, it is crucial that the capacity management plan has a reliable amount of bed capacity available to provide appropriate care to all patients in the area. The adult occupancy rate of GMC is frequently above 90% (well above national average).

Previous efforts at GMC focused on the development of a Monte Carlo simulation (MC) to study the relationship between the surgical schedule and crowding, by modeling length of stay and patient flow for surgical and non-surgical patients [18]. The results of the MC simulations were then reported and used by the Geisinger Placement Services (GPS) to predict overcrowding and make management decisions. When excessive bed demand is forecasted, the GPS team can decide to take actions including decanting surgical cases to nearby affiliated hospitals to perform non-urgent surgeries, reorganizing surgical case schedules, giving priority to expedited discharge processes, and adjusting staffing levels. The MC could accept user input to run what-if scenarios based on planned changes to surgical volumes at GMC to re-estimate inpatient bed demand. When surgeries are moved to nearby affiliated hospitals, the beds at GMC can be reallocated to meet the demand of the ED, OR and other arrival sources. This approach was able to capture the logic of the problem, but fell short in performance, making it necessary to revise the model.

To improve the performance of the forecast, a predictive modeling approach was proposed. A series of neural network-based models were built to predict from 1 to 5 days ahead, improving the performance obtained by the MC.

## Literature review

### Hospital census forecasting

The majority of work published about forecasting in hospitals relates to patient visits to the ED or other specific departments inside hospitals [13, 19–24]. An additional layer of complexity is added through the irregularity and volatility of in-patient visits when predictions are made for a hospital’s in-patient census. These predictions involve patients not just in the ED but in other departments which have different process flows, service times and lengths-of-stay (LOS). ML based models overcome this input complexity by using relevant factors as predictors [25].

The analysis from one research study identified that at an occupancy level of 100%, there is a 28% chance of at least one severe event occurring and a 22% chance of more than one severe event occurring [6]. Depending on the type of strategic decision-making time horizon, there are different models developed for predicting inpatient demand. Longer term strategy needs give rise to models that predict monthly forecasts [25]. More immediate strategies need to give rise to models with shorter horizons like the next 3, 5, or 7 days [24].

### Forecasting methods

There is a relatively small number of previous research developed in the context of bed demand forecasting [26], particularly using ML models. Simple autoregressive integrated moving average (ARIMA) models are among the first tools used to forecast bed demand [27]. One of the most common methodologies for estimating hospital bed demand is based on the valuation of the patient’s LOS at the hospital [28, 29]. Mackay et al. in 2005 proposed that these models are defective due to the complexity of the long-stay distribution, among other reasons. Hence, the authors investigated model selection and assessment in relation to hospital bed compartment flow models using The Bed Occupancy Management Planning System (BOMPS) software, in which hospital bed prediction models are developed from the simplest to the most complex, depending on their prediction horizon and model structure [30]. Ordu et al., in 2019 developed a framework for generating demand prediction models for each of the hospital areas [31]. The models are simple in nature and vary from ARIMA models to exponential smoothing, multiple linear regression (MLR), and seasonal and trend decomposition, where the prediction horizons are daily, weekly, or monthly depending on the hospital unit. The models proposed, for instance for Accident and Emergency admissions, have a low adjusted R^2^ value of 60%, where the monthly MLR produced the best goodness of fit. Kutafina et al. in 2019 developed a recursive neural network model for forecasting hospital bed demand occupancy (the most similar study to ours). In this article, authors propose different training alternatives for prediction models based on different time intervals for the training data set (from 1 to 5 year) and different dates and horizons for prediction [26]. Their results show an average mean absolute percentage error (MAPE) of 7.22% (5.76–9.22%) with a mean absolute error (MAE) of 15.65 beds for yearly predictions between 2009 and 2015. As discussed in^26^ the model that is proposed here (K-SVR) requires only historical admissions and no private/sensitive patient information (e.g., age or vital signs), as those models, for instance, try to infer the patient’s LOS. We show with our model that the decomposition of the historical demand provides a better means to generate accurate predictions of hospital bed demand for 1 and 2 days ahead.

## Methods

### Inpatient bed demand

We define inpatient bed demand as the number of patients occupying inpatient beds (census) plus the number of patients that should be in inpatient beds but are in another location (or in a holding pattern) such as the ED, PACU, or Cardiac Recovery Suite (CRS).

The population in this study included all adult patient encounters at GMC for 5 years. The data was complemented with 2 years of the most recent data. Patient and hospital data were collected using Geisinger’s electronic health record (EHR) and unified data-architecture (UDA). The data was then extracting by querying our data warehouse server and shared with our research partners via data use agreement. Our population included patients who had a patient class of inpatient, observation, or SORU at the time of their discharge. The population excluded patients who spent any time in a pediatric unit, had a level of care of psych, or did not have one of the following levels of care: critical care, step down, medical, surgical, telemetry, or surgical overnight.

### K-SVR forecasting model

**Fig. 1:**
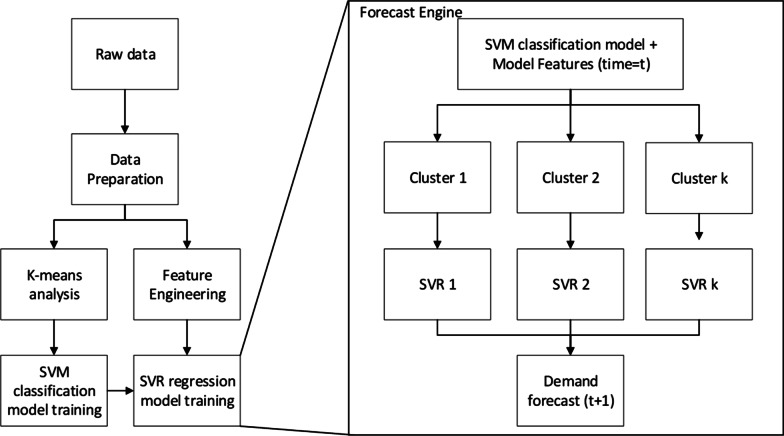
Schematic representation of the forecaster engine K-SVR and data preprocessing

The predictive strategy used in this study follows the model proposed by Feijoo, Silva and Das., (2016), which combines a classification stage followed by a regression (forecast) stage. The engine seeks to assign (classification stage) the response variable, in this case, future bed demands, into one of different groups or clusters of demands. The classification stage of the forecast engine is performed using a Support Vector Machine (SVM) model. Following the classification stage, for each of the clusters, a Support Vector Regression (SVR) model is locally developed, i.e., each cluster considers a unique forecasting regression model that seeks to accurately predict bed demands of days that follow similar historical patterns (forecast stage of the forecasting engine). Hence, the forecaster engine contemplates a set of “K” SVR models (K-SVR), where K represents the number of clusters to be considered. The cluster analysis and the subsequent selection of K (number of clusters) is based on the K-means clustering method; however, any other clustering approach could be used. A schematic representation of the forecaster engine is shown below in Fig. 1. As it can be noted, the algorithm starts with the data processing followed by the cluster analysis and the feature engineering step. As mentioned above, the clustering analysis guides the selection of K clusters, while the feature engineering step (based on partial and autocorrelation functions) allows to determine lagged values and seasonal patterns used in the regression stage (SVR model). Once K, lagged, and seasonal parameters are selected, we train an SVM model (classification stage) based on the clusters identified with the K-means method. For each of these clusters, a regression model, based on SVR, is trained, considering the lagged and seasonal parameters found in the feature engineering preprocessing step. Note that the framework is general enough to work as a simple 1-day ahead prediction, or for more complex tasks of recursively forecasting n-days ahead.

**Fig. 2:**
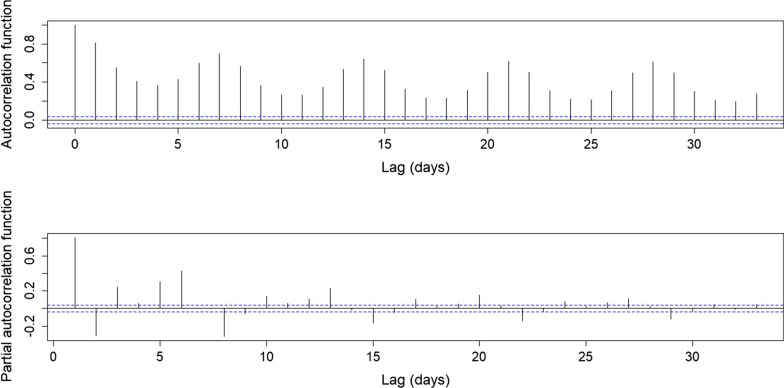
Autocorrelation and Partial autocorrelation functions results. Information used to select lagged values for K-SVR models

The SVR models are developed using a mixed-features a selection method, based on the information provided by the autocorrelation and partial autocorrelation functions (see Fig. 2 in results). These functions suggest the use of previous or lagged values for demand (previous days) and seasonal patterns for the model. On the other hand, an incremental strategy is simultaneously used for variable or feature selection, which refers to starting with an empty model and then adding potential predictors. As predictors are added to the model, this strategy seeks to explain whether and to what extent each predictor reduces unexplained variation. The list of final selected covariates used in the model, which provide the best forecast accuracy in the training process, is shown in Table 1. Hence, bed demand can be forecasted using time series information from previous demand days as well as other covariates’ historical information. Mathematically, the SVR model can be represented as a regression model as follows,

1 $${\widehat{Y}}_{t+1}=\sum_{i=0}^{l-1}{\beta }_{i}{Y}_{t-i}+\beta {Y}_{t-(s-1)}+\sum_{j=1}^{P}\sum_{i=0}^{l-1}{\beta }_{ij}{X}_{t-i}^{j}$$

where ${\widehat{Y}}_{t+1}$ represents the forecasted bed demand for 1 day ahead (t + 1), ${Y}_{t-i}$ represents the lagged values (l lagged values) of bed demand days, ${Y}_{t-(s-1)}$ accounts for the seasonal association (s denotes the lag for a seasonal trend), and ${X}_{t-i}^{j}$ considers every other covariate (P covariates) used for the prediction that is not a lagged value of bed demand (see Table 1 for a list of all covariates).

**Table 1:** Variable definition

Data set and variable definition	
Demand (Dt)	Present day demand data
Dt.1	Demand from one day ago
Dt.2	Demand from two days ago
Dt.3	Demand from three days ago
Dt.7	Demand from seven days ago
Dt.1.2	Interaction between Dt.1 and Dt.2
Mt	Yesterday Medicine Census
Ut	Yesterday Surgical Census
Cen_Mid	Yesterday’s total 12AM census

### Measures of forecast performance

**Fig. 3:**
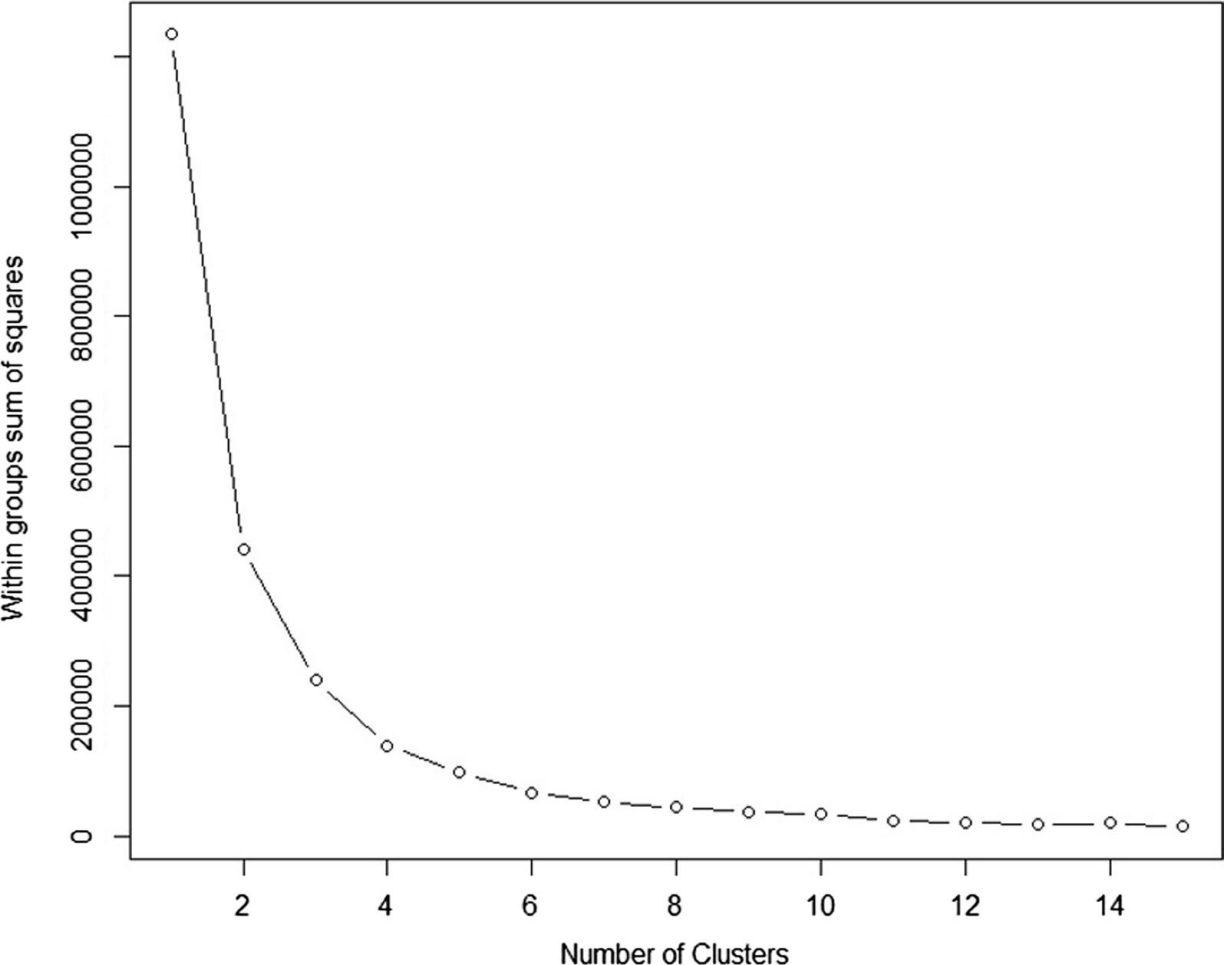
Within groups sum of square based on the number of clusters considered to group historical bed demand

The K-SVR model is developed and tested as follows. First, we chose 5 clusters to be used by the forecasting engine. The number of clusters is chosen based on minimizing the sum of squares within clusters (sum of cluster’s individual errors). It is important to balance between the number of clusters and the available data that falls within each cluster. A large number of clusters will significantly reduce the sum of squares within clusters, however, it is possible to obtain clusters with few data points, for which the local SVR model will tend to forecast overfitted values. Here, we made the selection of 5 clusters based on the errors shown in Fig. 3. The classification SVM layer as well as the local SVR models are created using 70% of the data for training purposes, and tested on specific dates (days, weeks) that fall within the testing period. The training and testing time series do not have any overlapped data points. Additionally, the model is tested (testing time series) on four independent test weeks (without overlapping). Also, both SVM and the SVR models were developed by optimizing (tuning) their input hyper-parameters and then trained with a tenfold cross validation. Finally, using the ACF and PACF information, we selected a lagged value (l parameter in Eq. 1) of 3 and a seasonal value (s parameter in Eq. 1) of 7. The model performance was tested using the following objective measures.

2 $$\mathrm{MAPE}=\frac{1}{\mathrm{N}}\sum_{\mathrm{i}=1}^{\mathrm{N}}\frac{\left|{\mathrm{Y}}_{\mathrm{i}}^{\mathrm{r}}-{\widehat{\mathrm{Y}}}_{\mathrm{i}}^{\mathrm{f}}\right|}{{\mathrm{Y}}_{\mathrm{i}}^{\mathrm{r}}},$$

3 $$\mathrm{MAE}=\frac{1}{\mathrm{N}}\sum_{\mathrm{i}=1}^{\mathrm{N}}\left|{\mathrm{Y}}_{\mathrm{i}}^{\mathrm{r}}-{\widehat{\mathrm{Y}}}_{\mathrm{i}}^{\mathrm{f}}\right|,$$

4 $$\mathrm{MSE}=\frac{1}{\mathrm{N}}\sum_{\mathrm{i}=1}^{\mathrm{N}}{\left({\mathrm{Y}}_{\mathrm{i}}^{\mathrm{r}}-{\widehat{\mathrm{Y}}}_{\mathrm{i}}^{\mathrm{f}}\right)}^{2}$$

The elements ${Y}_{i}^{r}$ and ${\widehat{Y}}_{i}^{f}$ in Eqs. (2)–(4) represent the real and forecasted bed demand, respectively. Equation 2 estimates the MAPE, defined as the average among the absolute value of the error forecast over the real bed demand (average error of the forecast model), where N forecast have been performed. The MAE is shown in Eq. 3. The MAE provides the average absolute error in terms of beds being missed forecasted by the model. Finally, we use the standard Root Mean Square Error (RMSE = $\sqrt{MSE}$) as a third metric to measure for error variability.

## Results

We now present the results from the model on distinct instances. First, we developed a model to forecast the bed demand for 1 day ahead while considering patients coming into the ED during the weekends. This approach helps to better correlate the data, even though there are no surgeries scheduled on those days. As a counterpart case, we present a model that only uses weekday (Monday to Friday) data, hence creating a different lagged pattern on the model. We then develop and present the results for a 2 day ahead bed demand prediction following the same approach data availability approach (weekends and week only data).

### Data set statistical description

The K-SVR model is built based GMC data corresponding to all adult patient encounters at GMC for 5 years. The specific variables obtain from the data and used to create the K-SVR model are described next.

**Fig. 4:**
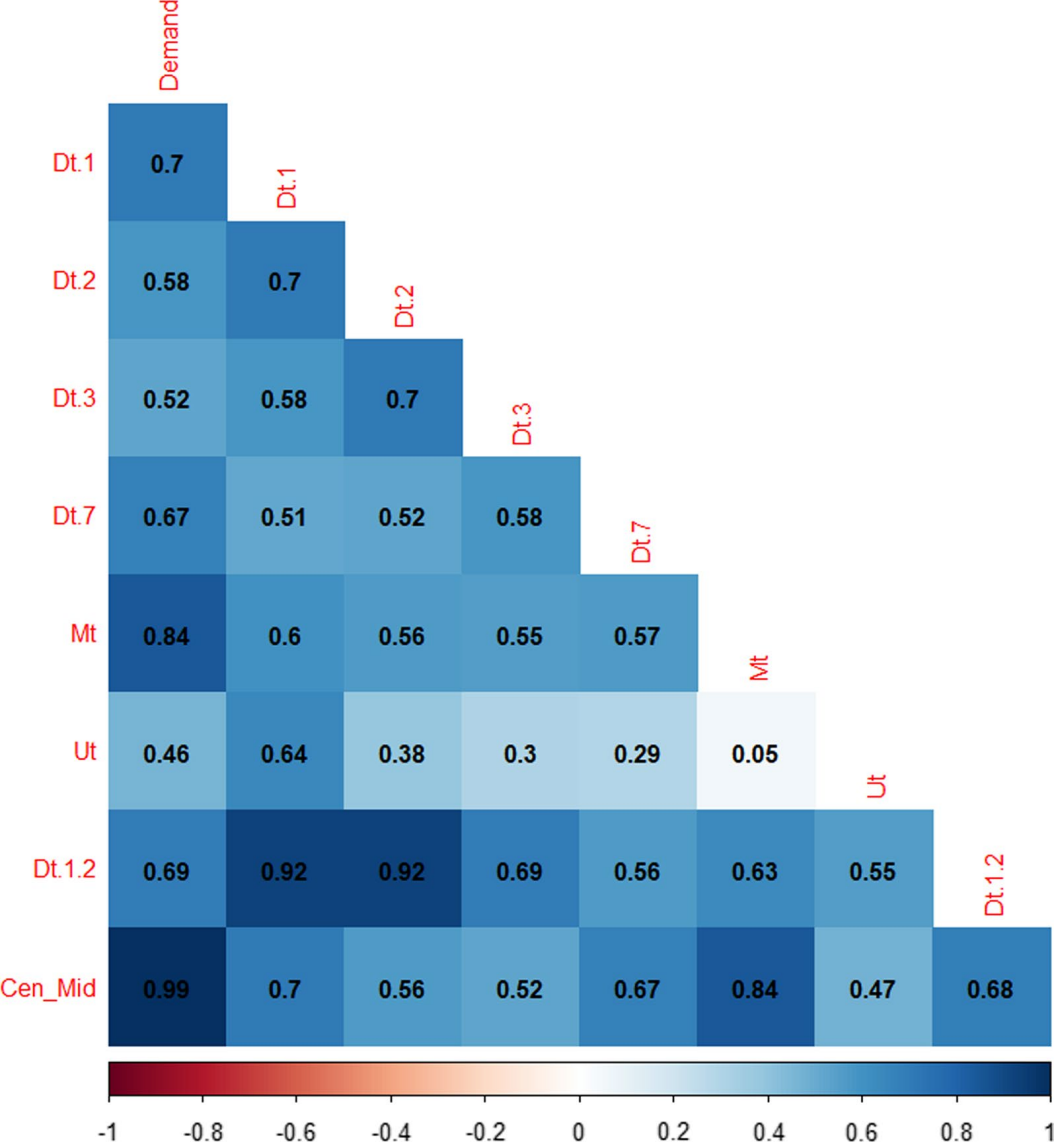
Correlation Matrix for data used in the forecast model

Table 1 shows the variable definition. The data considers historical utilization of beds (demand). We used lagged values (3 days) and a weekly seasonal lag (7 days) of demand. Such lags were obtained based on the ACF and PACF functions, as shown in Fig. 2. The significance of the lagged values can also be observed on the correlation matrix as shown in Fig. 4.

We develop four different models, each corresponding to different forecasting timeframes (1 day and 2 days ahead, with and without weekends data). For all four models, we used a number of K = 5 clusters. This number is obtained based on the within-groups sum of squares results shown in Fig. 3. From the figure we observe that for a number of clusters (K) larger than 5, the reduction of the sum of squares (error or differences within clusters) is small. Also, it is important to note that a larger value of K can be chosen if variance within clusters wants to be reduced. However excessively reducing variance on each clusters may result in subsets with a small number of data points, increasing the risk of developing overfitted models. Therefore, using the standard “elbow” method in clustering analysis, K = 5 is chosen. Finally, all models are tested on four non-consecutive different test weeks (same weeks for each of the four K-SVR models developed) that do not belong to the training time series data. Results of the K-SVR models are compared to an autoregressive integrated moving average (ARIMA) model, as data satisfies the assumptions for such models, as well as a version of K-SVR with K = 3 (as compared to K = 5), to demonstrate the fact that lower values of K in fact increase the variability within each cluster, increasing the forecast error.

### Prediction of bed demand for a day considering weekends

**Fig. 5:**
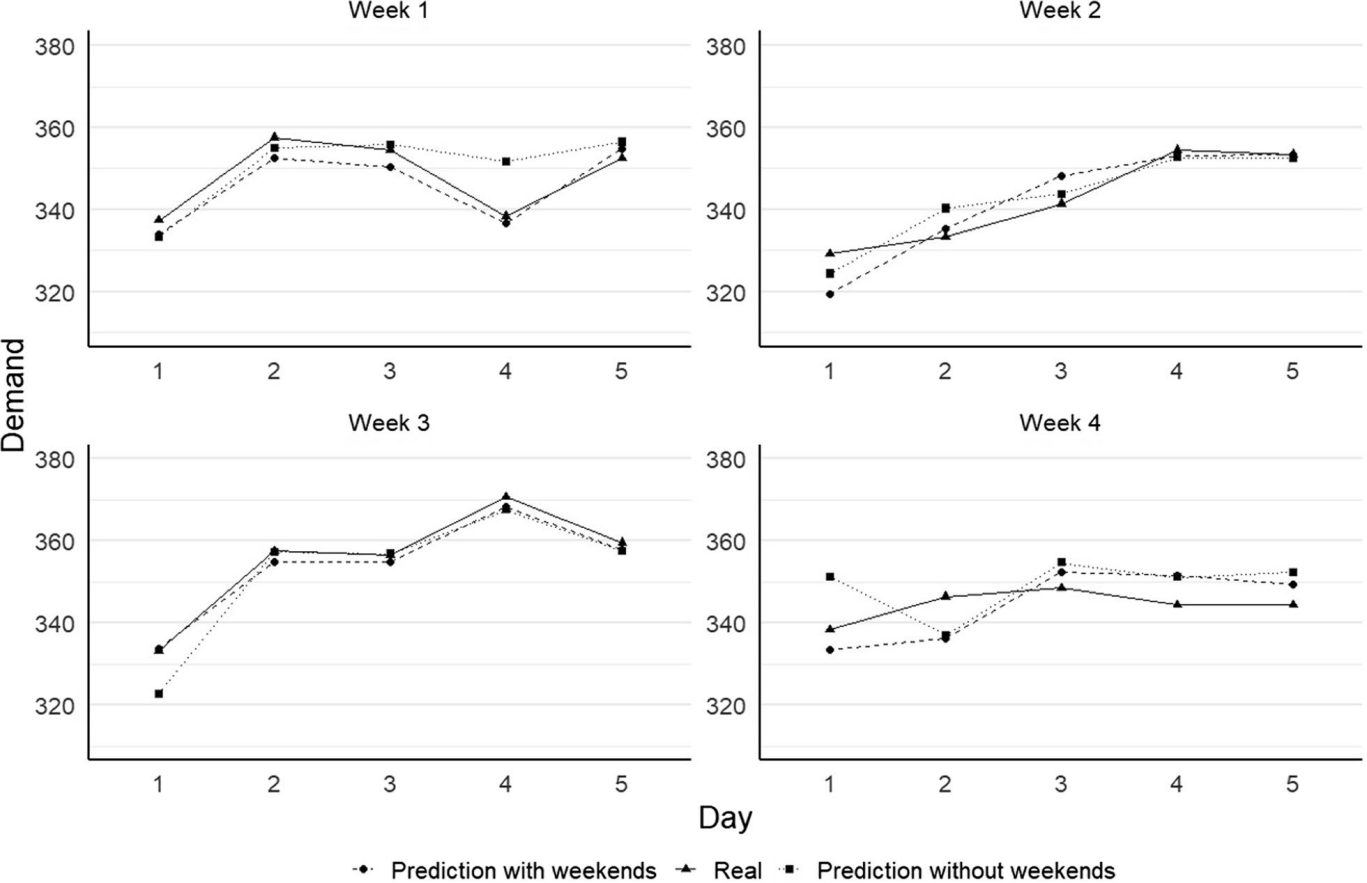
Forecast results from the K-SVR engine for one day ahead considering with and without weekends for four distinct sample weeks

As previously mentioned, an initial model with K = 5 was developed to generate a prediction for 1 day ahead while using weekend data. The optimal K-SVR model is compared against an ARIMA model and a different version of K-SVR with lower number of clusters (non-optimal). The performance of all models was evaluated based on the MAPE, MAE, RMSE and error variance, as shown in Table 2. The average bed demand error (MAE) for the days on which the K-SVR (K = 5) model was tested (random weeks on the test data, not overlapping with time series used for training of the models), as indicated in the last column, shows a mean absolute deviation of 3.81 beds per day with a standard deviation of 4.36 beds, representing an average percentual error of 1.11%. The K-SVR model with K = 3 obtained an average (across the four test weeks) MAPE of 1.35%, while the ARIMA model resulted in an average MAPE of 3.29%. When analyzing the test week individually (not average), we obtain MAPE results as low as 0.49, showing the high accuracy that the optimal K-SVR model can obtain. For this week, the mean error is of 1.76 beds with an RMSE of 1.90. The test week 4 shows the lowest (but still accurate) performance, with a MAPE of 1.81%, resulting in mean absolute error of 6.24 with an RMSE of 6.64. Figure 5 shows the high precision of the forecast results for the four test weeks.

**Table 2:** Summary of the results (performance measures) for K-SVR model for 4 random test weeks

Metric	Model	Week 1	Week 2	Week 3	Week 4	Average
*1-day ahead with weekend data*						
MAPE (%)	K-SVR	0.93	1.19	0.49	1.81	1.11
	K-SVR(3)	0.76	1.49	0.73	2.40	1.35
	ARIMA	3.45	2.64	2.86	4.22	3.29
MAE (bed/day)	K-SVR	3.25	4.01	1.76	6.24	3.81
	K-SVR(3)	2.69	5.03	2.65	8.30	4.67
	ARIMA	11.89	8.92	10.19	14.47	11.37
RMSE (bed/day)	K-SVR	3.46	5.45	1.90	6.64	4.36
	K-SVR(3)	3.15	6.31	3.32	9.08	5.98
	ARIMA	13.68	9.87	10.55	15.27	12.34
Error variance	K-SVR	1.00E−05	1.20E−04	0.00E+00	4.00E−05	5.00E−05
	K-SVR(3)	2.12E−05	1.27E−04	2.86E−05	1.14E−04	7.27E−05
	ARIMA	5.16E−04	2.05E−04	6.67E−05	2.68E−05	2.04E−04
*1-day ahead with weekend data*						
MAPE (%)	K-SVR	2.55	1.02	0.88	2.65	1.78
	K-SVR(3)	1.85	0.92	0.65	3.39	1.70
	ARIMA	3.42	2.05	2.96	4.00	3.11
MAE (bed/day)	K-SVR	8.91	3.45	3.13	9.11	6.15
	K-SVR(3)	6.33	3.19	2.30	11.65	5.87
	ARIMA	11.69	7.08	10.41	13.75	10.73
RMSE (bed/day)	K-SVR	11.74	4.33	3.93	9.65	7.41
	K-SVR(3)	8.11	3.61	3.01	12.05	7.63
	ARIMA	14.56	10.06	12.40	15.22	13.06
Error variance	K-SVR	4.60E−04	1.00E−05	4.00E−05	9.00E−05	1.50E−04
	K-SVR(3)	2.34E−04	2.91E−05	3.12E−05	9.25E−05	9.67E−05
	ARIMA	8.35E−04	5.07E−04	4.78E−04	4.58E−04	5.69E−04

### Prediction of bed demand for 1 day ahead without considering weekends

We tested the two K-SVR (K = 5 and K = 3) and the ARIMA models on the same four sample weeks as in "Data set statistical description" section. where data that is available during the weekend days is removed from the model. For instance, predictions for the first day of the week, Monday, is made based on the information available on the Friday from the corresponding previous week. Thereafter, since this is a 1 day ahead prediction, forecasts for Tuesday are made based on data from the Monday of the same week and the lagged value 2 would correspond to Friday from the previous week. The same logic applies for the lag values 3 and 7 and the remaining days of the test weeks (Wednesday to Friday). Results of the models for the 4 performance measures are also shown in Table 2. It is possible to observe that, on average, the optimal K-SVR model obtains a MAE of 6.15 beds per day, with a RMSE of 7.41 beds per day, representing an average percentual error of 1.78%. As in the previous case, the best performance was obtained for the test week 3, with a MAPE value of 0.88%, representing a MAE of 3.13 beds per day with a RMSE of 3.93 beds per day. Figure 5 shows the comparison between the forecasted values and the actual values for each of the test weeks. For this case, opposite to the case where weekend data is available and for two-days ahead forecast (see next subsections), the two K-SVR models showed similar results, while the ARIMA model was again the least accurate.

Generally, when compared to the previous case (1 day ahead with weekend data), we observed that the forecast accuracy slightly drops. Among the 4 weeks, the average MAPE for this case is 1.78%, compared to 1.11% when weekend data is considered, representing a 0.67% difference. In absolute terms, the difference in the (average) MAE is 2.34 beds (3.81 versus 6.15 when weekend is considered) with a RMSE difference of 3.05 beds per day (7.41 compared to 4.36). Depending on the data access and availability, the small differences between the two models allows for reliance on both models. The forecast comparison for both previous models (with and without considering weekend data) and the real demand, for the four random test weeks, are presented in Fig. 5.

### Prediction of two-day bed demand considering weekends

In this section we present and explain the results for the 2 day ahead forecast model. The model characteristics are the same as those presented earlier, with the consideration that the forecast is made for 2 days ahead. Hence, proper data manipulation must be considered to correctly define the lag-values that are used as inputs for the model. We first present the results for the 2 day ahead model with the consideration of the weekend data for all three models (two K-SVR and the ARIMA model).

**Fig. 6:**
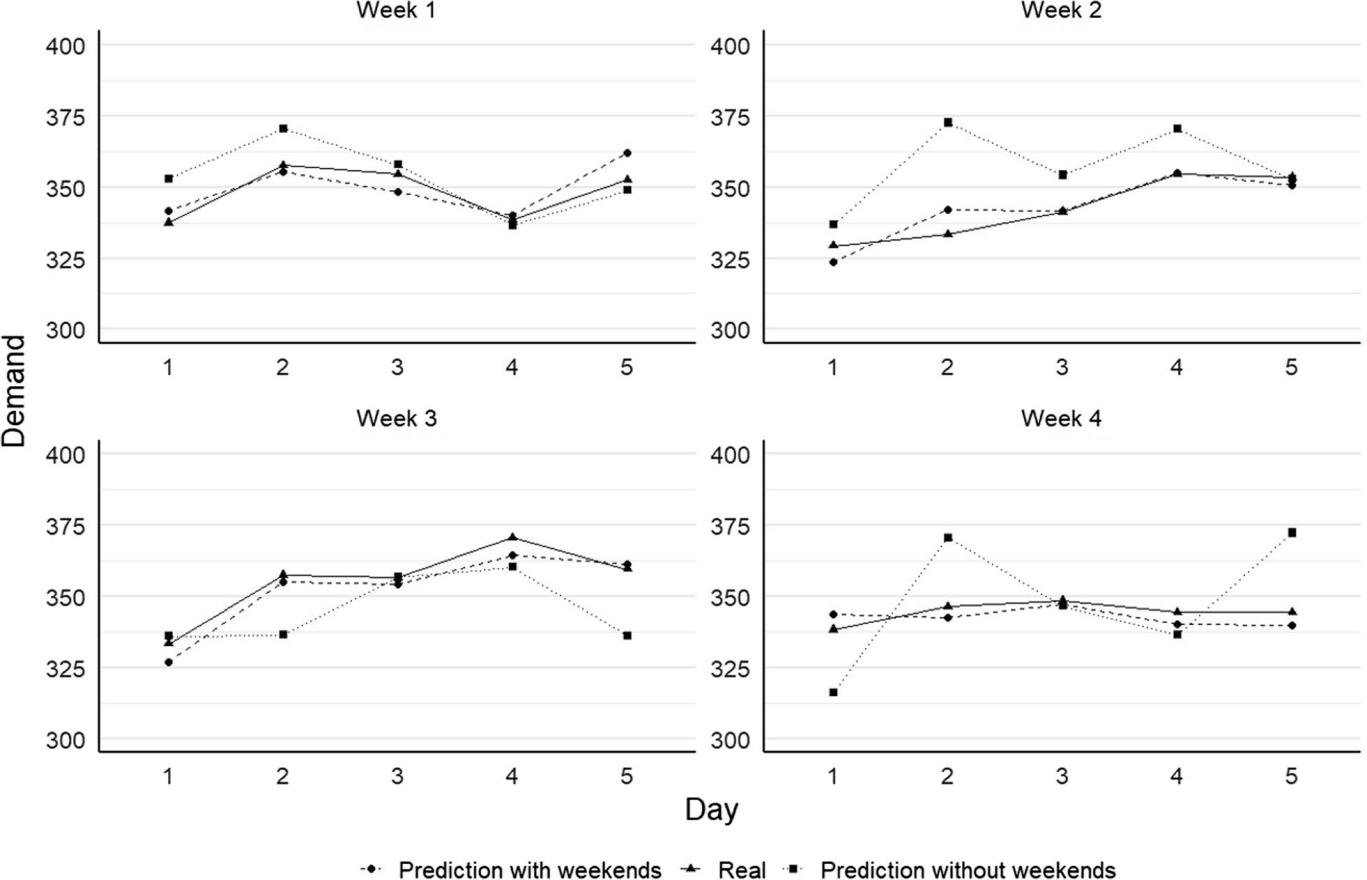
Forecast results from the K-SVR engine for 2 day ahead considering with and without weekends for four distinct sample weeks

Table 3 shows the results for the scenario described above and for four sample weeks used to test the 2 day ahead model. We obtain, on average among the 4 weeks, a MAE 4.89 beds per day, with a RMSE of 5.77 beds per day, which translates to an MAPE of 1.42% (optimal K-SVR). Note that, for a fair comparison in this case, we consider the same four random test weeks as in the case for 1 day ahead. Figure 6 shows the result of the forecasted demand and the actual values for each of the test weeks. Interestingly, when weekend data is considered, the optimal K-SVR model behaves similarly as the case of the 1 day ahead model. The lowest MAPE values were obtained for the sample week 3, followed by week 2. As previously noted, the ARIMA model again shows the least accurate results, while the K-SVR with K = 3 performs better than ARIMA but not as accurate as the case of K-SVR with 5 clusters.

**Table 3:** Summary of the results (performance measures) for the 2-day ahead K-SVR model for 4 random test weeks

Metric	Model	Week 1	Week 2	Week 3	Week 4	Average
*2-day ahead with weekend data*						
MAPE (%)	K-SVR	1.41	1.07	0.58	2.62	1.42
	K-SVR(3)	1.83	1.40	0.83	2.62	1.67
	ARIMA	3.87	2.62	8.25	3.13	4.47
MAE (bed/day)	K-SVR	4.87	3.61	2.08	9.01	4.89
	K-SVR(3)	6.33	4.75	2.94	8.98	5.75
	ARIMA	14.15	9.61	26.42	10.84	15.26
RMSE (bed/day)	K-SVR	6.88	4.51	2.36	9.32	5.77
	K-SVR(3)	7.74	5.06	3.39	9.92	6.99
	ARIMA	16.98	10.49	29.04	13.26	17.44
Error variance	K-SVR	2.10E−04	6.00E−05	0.00E+00	5.00E−05	8.00E−05
	K-SVR(3)	2.63E−04	6.13E−05	4.56E−05	2.76E−04	1.61E−04
	ARIMA	7.51E−04	1.56E−04	2.12E−03	5.91E−04	9.04E−04
*2-day ahead without weekend data*						
MAPE (%)	K-SVR	3.18	4.10	2.75	3.33	3.34
	K-SVR(3)	6.21	4.32	6.17	5.98	5.67
	ARIMA	3.97	3.16	9.75	3.80	5.17
MAE (bed/day)	K-SVR	11.08	13.91	9.89	11.46	11.59
	K-SVR(3)	21.72	14.74	21.65	20.56	19.67
	ARIMA	14.41	11.32	30.98	12.97	17.42
RMSE (bed/day)	K-SVR	12.39	17.84	12.79	13.38	14.10
	K-SVR(3)	29.48	17.69	25.68	25.51	24.96
	ARIMA	16.73	12.43	38.59	14.52	20.57
Error variance	K-SVR	2.60E−04	1.12E−03	5.10E−04	4.00E−04	5.70E−04
	K-SVR(3)	3.02E−03	9.51E−04	1.43E−03	1.79E−03	1.80E−03
	ARIMA	6.41E−04	2.82E−04	7.28E−03	4.88E−04	2.17E−03

### Prediction of bed demand for 2 days without considering weekends

Lastly, we use both K-SVR and the ARIMA models to forecast 2 days ahead without considering weekend data. We use the same four sample weeks as in the previous three cases. As in the case for 1 day ahead, the data that can be available during weekend days is considered to fit the K-SVR model. Hence, the bed demand prediction made for the first day of the week, Monday, considers information available on the Thursday from the corresponding previous week (the real prediction is 4 days ahead when no weekend data is considered). Since this is a 2 day ahead prediction, the forecast for Tuesday is made based on data from the Friday of the previous week. This process is repeated until the forecast for the whole test week is completed. The same logic applies for the lag values 2, 3, and 7 and the remaining days of the test weeks (Wednesday to Friday). Results of the models for the 4 performance measures are shown in Table 3. It is possible to observe that for the optimal K-SVR, on average, the MAE corresponds to 11.59 beds per day, with an RMSE of 14.10 beds per day, representing a MAPE of 3.34%. As in the previous case, the best performance was obtained for test week 3, with a MAPE value of 2.75%, representing a MAE of 9.89 beds per day with an RMSE of 12.79 beds per day. Similarly, to previous cases, the alternative K-SVR (K = 3) and the ARIMA model do not achieved the accuracy levels than the best K-SVR (K = 5) model. In this particular case, when considering 3 clusters, K-SVR even performs similarly to the ARIMA model. Figure 6 shows the comparison between the forecasted values and the actual values for each of the test weeks. To facilitate the comparison, Fig. 6 also shows the results for 2 day ahead with weekend data. It is clearly observed that the results for 2 day ahead model without weekends (i.e., 4 days ahead for the first 2 days of a week) are the less accurate among all the models and cases presented here.

When we compare the two cases (with and without weekends) for a 2 day ahead forecast (see Fig. 6) using the K = 5 SVR model, we observed that the forecast accuracy drops significantly for the latter case. Among the 4 weeks, the average MAPE for this case is 3.34%, compared to 1.42% when weekend data is considered, representing a 1.92% difference between these two cases. In absolute terms, the difference in the (average) MAE is of 6.7 beds per day (11.59 versus 4.89 when weekend is considered).

**Fig. 7:**
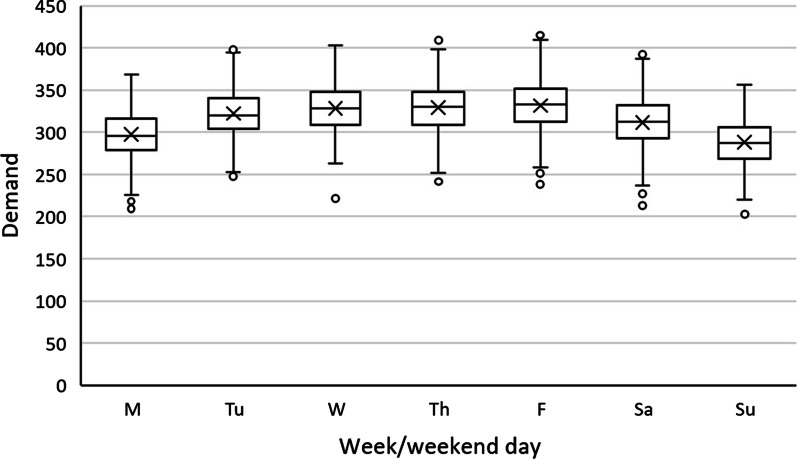
Demand by weekday/weekend day

The reasons for the model to perform better with weekend data are twofold. First, as seen in Fig. 7 below, typically the census drops on the weekend after mid-week highs. This is primarily due to more scheduled surgeries and physician availability during the weekdays. Leaving this data out is analogous to leaving a part of the trend out from the model. For example, the lows on Sunday might play an important role on the rate of increase of census Monday through Wednesday. Including weekend data would help identify these signals. Secondly, in our analysis we saw the interactions between the previous few days as significant. Excluding weekend data would mean that we are excluding these interactions of the past few days. Excluding weekends and predicting for Monday using last Friday’s data is similar as predicting 3 days ahead instead of 1 day ahead. The further into the future we forecast, the further the prediction accuracy is expected to drop. This drop in performance would also explain the performance difference between the models that used weekday and weekend data.

## Discussion and conclusions

While hospitals in general would like to reduce occupancy rates to improve patient outcomes, hospitals also need to proactively plan on having enough capacity and improve processes in order to provide timely care for patients to improve patient wait times and satisfaction. Additionally, maintaining staffed beds is expensive and challenging, therefore, hospitals have an economic incentive to maintain high utilization without running out of capacity. When hospital operations decision makers have easy access to near term bed demand forecasts, combined with their expertise and real time assessment of their bed capacity, they can make proactive, data driven operational decisions. Inputs to these models consist of daily updated time-series data which is captured in our hospital’s UDA. Once the model is trained, we deploy it on our servers where it makes daily forecasts with updated data. These forecasts can be made accessible to key hospital operations decision makers through interactive dashboards or other modes of communication.

The MAE and MAPE performance for the models presented in this paper are within acceptable parameters to hospital administration stakeholders, allowing them to make data driven decisions. When bed demand forecasts are high, they can begin expediting low acuity patient discharges, decant elective surgical cases to nearby affiliate hospitals or reschedule them, and mandate nursing overtime one to 3 days in advance to prepare for the increased bed demand. Mandating nursing overtime or increasing nursing staffing days in advance eliminates the need to rely on expensive, contracted nursing and support staff. Extra bed capacity can then be used to handle the increased patient demand, allow appropriate patient flow in accordance with their level of care, and minimize patient holding times in the PACU, the ED, and other patient arrival sources. When forecasts point to lower bed demand, stakeholders can ensure hospitals aren’t overstaffed and potentially increase patient satisfaction with private patient rooms. We currently predict the adult census for the entire hospital and don’t delineate by level of care. In the future, with access to additional granular department level data, we can extend the models presented in this paper to get detailed forecasts for individual departments within a hospital. Considering what’s currently been seen with the COVID-19 pandemic, this model, with additional COVID-19 hospitalization data, could be used to help manage hospital capacity in uncertain times. Now that COVID-19 will be a part of every hospitals’ bed demand, advanced bed demand models will be necessary more than ever to quantify and forecast hospital capacities for a variety of scenarios including surges and shut-downs.

## Data Availability

The datasets generated and/or analyzed during the current study are not publicly available because they contain business sensitive information. Data could be requested from Geisinger with a basic data use agreement. Requests can be started with an email to the authors or Geisinger’s Steele Institute for Health Innovation (SteeleInnovation@geisinger.edu).

## Abbreviations

ML: Machine learning
K-SVR: K-means support vector regression
SVM: Support vector machine
GMC: Geisinger medical center, Danville
MC: Montecarlo simulation
ED: Emergency department
PACU: Post-acute care unit
SORU: Surgical overnight recovery
MAPE: Mean average percentage error
GPS: Geisinger placement team
LOS: Length of stay
ARIMA: Auto regressive integrated moving average
MLR: Multiple linear regression
EHR: Electronic health record
UDA: Unified data architecture
CRS: Cardiac recovery suite
ACF: Auto-correlation function
PACF: Partial auto correlation function
MAE: Mean absolute error
RMSE: Root mean squared error
